# Assessment of the main signaling pathways involved in the combined therapy of hepatocellular carcinoma using Sorafenib and NK cells in xenograft mice model

**DOI:** 10.1016/j.toxrep.2025.102131

**Published:** 2025-09-22

**Authors:** Faezeh Hosseinzadeh, Tahereh Komeili movahhed, Masoumeh Dolati, Amir Hossein Kheirkhah, Nima Beheshtizadeh, Javad Verdi

**Affiliations:** a Department of Tissue Engineering and Applied Cell Sciences, School of Medicine, Qom University of Medical Sciences, Qom, Iran; b Cellular and Molecular Research Centre, Qom University of Medical Sciences, Qom, Iran; c Clininal Trial Center, Qom University of Medical Sciences, Qom, Iran; d Student Research Committee, Qom University of Medical Sciences, Qom, Iran; e Department of Tissue Engineering, Faculty of Advanced Medical Sciences, Tabriz University of Medical Sciences, Tabriz, Iran; f Regenerative Medicine Group (REMED), Universal Scientific Education and Research Network (USERN), Tehran, Iran; g Department of Applied Cell Sciences, School of Advanced Technologies in Medicine, Tehran University of Medical Sciences, Tehran, Iran

**Keywords:** Hepatocellular carcinoma, Sorafenib, Natural killer cells, Signaling pathways

## Abstract

Hepatocellular carcinoma (HCC) is the third leading cause of cancer-related deaths worldwide. Sorafenib is the only FDA approved drug for HCC patients, affecting patient survival by just a few months with significant toxicities in approximately half of HCC patients and the poor prognosis of these patients. Thus, the combination therapy with Sorafenib and Natural killer (NK) cells has been suggested, due to NK cells' distinct effectiveness against HCC. This research examined the main signaling pathways in HCC progression affected by Sorafenib or NK cells in combination or individual treatment. The xenograft model of HCC was created by implanting human HepG2 cells subcutaneously into the flank of 12 nude mice and then divided into four groups: Control, Sorafenib, NK cells, and Sorafenib plus NK cells. Four weeks post tumor implantation, the mice were euthanized, and the levels of liver and kidney enzymes were analysed for safety purposes. Quantitative real-time PCR analysis was used to measure the expression of key effector genes related to Sorafenib and NK cell functions with focused on the signaling pathways involved in the development of HCC. The levels of Aspartate aminotransferase (AST), Alanine transaminase (ALT), Blood urea nitrogen (BUN), and creatinine (Cr) in all groups remained in normal ranges. The expression levels of certain proliferative, anti-apoptotic factors were reduced in groups treated with either Sorafenib or NK cells only, but in combinational treated group showed no significant differences compared to control group. Also, the NK cell effector function related genes were upregulated in NK cell treated group, but inhibited in co administration of both NK cell and Sorafenib. Therefore, combining Sorafenib and NK cells at the prescribed dosage led to a decrease in the anti-cancer efficiency of both and may not be a successful option for HCC treatment.

## Graphical Abstract

**Figure:**
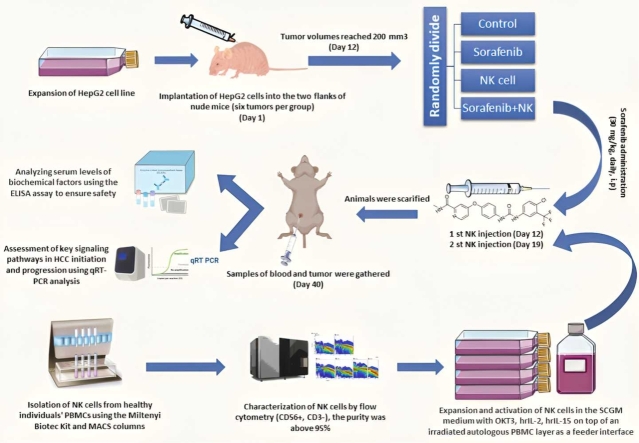

## 1 Introduction

HCC is extensively researched liver cancer and ranks as the third leading cause of cancer-related mortality, causing more than 800,000 deaths worldwide annually [1], [2]. The World Health Organization (WHO) predicts that over 1 million individuals will succumb to liver cancer by 2030 [3]. American Association for the Study of Liver Diseases (AASLD) in its 2018 recommendations for HCC management, advises monitoring for HCC in adults with cirrhosis, as it enhances overall survival. Tyrosine kinase inhibitors (TKIs) such as lenvatinib and Sorafenib, along with immunotherapy and anti-angiogenic treatments, provide additional therapeutic choices for advanced HCC [4]. A high rate of HCC-related death emphasizes the importance of developing new drugs or improving existing ones. Prior research demonstrated that numerous pathways, including the receptor tyrosine kinase (RTK) pathways, the phosphatidylinositol 3-kinase/protein kinase B (PKB), also known as AKT/mammalian target of rapamycin (PI3K/AKT/mTOR), the rat sarcoma virus/rapidly accelerated fibrosarcoma/mitogen-activated protein kinase/extracellular signal-regulated kinase (Ras/Raf/MAPK/ERK), the Janus kinase/signal transducer activator of transcription factor (JAK/STAT) and Wnt/β-catenin, are crucial in the advancement and propagation of HCC.

Multiple genes are regulated by the RTK downstream pathways and play critical roles in tumor development and progression. These pathways control the expression of key genes related to apoptosis, such as baculoviral inhibitor of apoptosis repeat-containing 5 (BIRC5), Cyclin D1 (CCND1), Nuclear factor-kappa B (NF-κB) and MYC proto-oncogene. Survivin, also known as BIRC5, is a part of the inhibitor of apoptosis protein family and is commonly found in high levels in HCC. It can prevent cell death by inhibiting caspase 3, 7, and 9, leading to excessive cell growth. The CCND1 gene encodes the protein cyclin D1, which plays a role in advancing the cell cycle by activating cyclin-dependant kinase. Cyclin D1 is a cell cycle regulator that activated by the PI3K/AKT/mTOR pathway and promotes progression through the cell cycle and its overexpression can lead to uncontrolled cell proliferation. MYC is a transcription factor activated by multiple pathways, including Ras/Raf/MEK/ERK and PI3K/AKT/mTOR, which promotes cell growth, proliferation, and metabolism, and its overexpression is a hallmark of many cancers. A growth factor that promotes angiogenesis, the formation of new blood vessels, VEGF, is often overexpressed in tumors due to activation of various pathways, including PI3K/AKT/mTOR and Ras/Raf/MEK/ERK, driving tumor growth and metastasis [5], [6].

The progression of HCC tumors significantly initiated by RTKs such as vascular endothelial growth factor receptor (VEGFR), platelet-derived growth factor receptor (PDGFR), epidermal growth factor receptor (EGFR), fibroblast growth factor receptor (FGFR), hepatocyte growth factor receptor (HGFR/ c-MET), stem cell growth factor receptor (c-KIT), and FMS-like tyrosine kinase 3 (FLT3) [7]. These RTKs, when abnormally activated, contribute to cancer cell proliferation, survival, angiogenesis (new blood vessel formation), and metastasis. Targeting these RTKs with specific inhibitors is a promising therapeutic strategy for HCC. When RTK binds to its ligand, it results in the phosphorylation of tyrosine on the target protein, which controls various signaling pathways like PI3K/AKT/mTOR, Ras/Raf/MEK/MAPK, JAK/STAT and Wnt/β-catenin, each with distinct biological functions [4], [8]. Dysregulation of these pathways, often through mutations in RTKs or downstream components, is a hallmark of cancer, contributing to uncontrolled cell growth and metastasis.

The PI3K/AKT/mTOR pathway activates by various growth factors and promotes cell survival, proliferation, and growth by inhibiting apoptosis and stimulating protein synthesis. PI3K phosphorylates PIP2 to PIP3, which recruits and activates AKT. Activated AKT, in turn, phosphorylates and inactivates TSC2, a negative regulator of mTOR. Dysregulation of this pathway is a frequent event in cancer, driving tumor growth and resistance to therapy [9].

The Ras/Raf/MEK/ERK pathway is essential for regulating cell growth, blood vessel formation, and changes in liver cells caused by growth factors, making it a possible treatment target for HCC. RTKs activate Ras, which recruits and activates Raf. Then activated Raf phosphorylates and activates MEK, which in turn activates ERK. Activated ERK translocates to the nucleus and phosphorylates various transcription factors, leading to changes in gene expression. Mutations in Ras, Raf, or MEK are common in cancer, driving uncontrolled cell proliferation. In this route, STAT3 is the key regulator for the transformation induced by RAS oncogenes. The transcription factor STAT3, which has been widely researched, is highly activated in HCC patients either directly by different cytokines and growth factors, or indirectly by the activation of the Ras/Raf/MEK/ERK pathway [5], [7], [10]. The STAT3 transcription factor is also activated by the JAK/STAT pathway. The JAK/STAT pathway is activated by cytokines and growth factors. RTKs can activate JAKs, which then phosphorylate STAT proteins. Phosphorylated STATs dimerize and translocate to the nucleus, where they regulate gene expression. The STAT family includes STAT1, STAT3, STAT5, and others, each regulating different sets of target genes [11], [12], [13].

The Wnt/β-catenin pathway also contributes to at least one third of HCC tumor advancement. The Wnt/β-catenin pathway is essential for regulating cell cycle and cancer development in both normal and abnormal cell functions. An increase in cytosolic β-catenin levels is triggered by the binding of Wnt ligands to Frizzled receptors on the surface of HCC cells, resulting in the activation of this pathway. The β-catenin subsequently translocates to the nucleus, inducing the transcription of genes such as Cyclin D1, c-myc (MYC), and Survivin that play a role in cell growth [5], [14]. Survivin and cyclin D1 are both indirectly activated by a transcription factor called octamer-binding transcription factor-4 (OCT4). Targeting both OCT4 and survivin is believed to be advantageous in the treatment of HCC [15], [16].

**Fig. 1:**
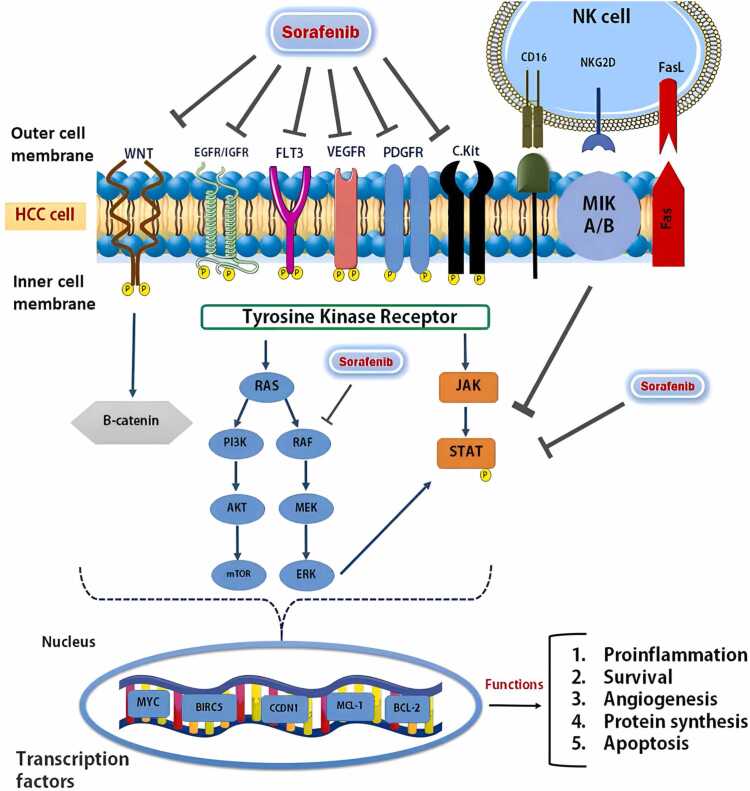
The schematic diagram of the key signaling pathways in liver cancer initiation and progression affected by Sorafenib or NK cells.

The sole FDA-approved drug for advanced HCC is Sorafenib, which functions as a multikinase inhibitor that hinders tumor growth and blood vessel formation. Sorafenib works as an anticancer agent by blocking RTKs like PDGFR, VEGFR, FLT-3, and cKIT, resulting in suppression of downstream signaling in the Ras/Raf/ERK and PI3K/AKT/mTOR pathways [17], [18]. Another way Sorafenib works is by directly stopping the tumor cells from multiplying and forming new blood vessels. This is achieved by inhibiting the Raf, which in turn reduces the phosphorylation, activation, and movement of STAT3 into the nucleus, ultimately leading downregulation of cyclin D1 [19], [20]. Moreover, Sorafenib can block the phosphorylation and activation of STAT3 (pSTAT3) by enhancing the levels of tyrosine phosphatase enzymes of SH2 domain-containing phosphatases 1 and 2 (SHP-1 and SHP-2). The suppression of the STAT3 pathway by Sorafenib involves the regulation of various downstream proteins like surviving, cyclin D1, Bcl-2 and Mcl-1, impacting cell proliferation and apoptosis [20]. Moreover, Sorafenib has the potential to function as an inhibitor of the Wnt/β-catenin pathway, which is excessively active in different cancers like HCC, through decreasing the levels of β-catenin and its buildup in the nucleus, ultimately resulting in the inhibition of its target genes (Fig. 1) [21], [22].

While Sorafenib has positive outcomes for advanced HCC patients, it only extends survival by less than 3 months and leads to significant toxicities in approximately half of patients [23]. A phase III clinical trial revealed that the typical lifespan with Sorafenib is 12.3 months [24]. Furthermore, additional research has indicated that resistance to Sorafenib reduces its efficacy in the treatment of HCC. Sorafenib resistance may result from the excessive activation of different pathways such as JAK/STAT and PI3K/AKT/mTOR, epigenetic modifications including noncoding RNA and methylation, induction of hypoxia-related pathways, immune environment, and epithelial-mesenchymal transition [25]. Hence, combinational therapies were proposed to find a practical approach to overcome the resistance to Sorafenib, including the combination of molecularly targeted agents, cytotoxic chemotherapeutic agents, targeting PI3K/AKT pathway, and immunotherapeutic strategy [26]. Recent studies exhibited that immunotherapy along with Sorafenib is more effective than Sorafenib alone. For instance, lo *et al.* showed that Sorafenib combined with an anti-CD47 antibody had a synergistic suppression of tumor growth in mice [27].

Another debatable choice is the use of a combination of Sorafenib and NK cells, due to their important role in the early immune response to HCC and their ability to kill different viral and cancer cells. NK cells are a type of lymphocyte with the ability to kill infected cells and boost immune responses against cancer cells. NK cells, as key components of the innate immune system, play a crucial role in anti-tumor immunity [28]. NK cells have the ability to induce cell death through the activation of the extrinsic apoptosis pathway with Fas/FasL, producing pro-inflammatory cytokines, and releasing granzyme and perforin during degranulation. Moreover, activating receptors such as NKG2D plays a crucial role for NK cell, boosting their cytotoxicity against HCC cells by blocking STAT3 when binding with MICA/B [29], [30] (Fig. 1). However, the success of adoptive transfer immunotherapy may be obstructed by the limited number and migration efficiency of NK cells, as well as the inhibitory conditions within tumors and the decreased cytotoxicity and cytokine generation of worn-out NK cells. It has been stated that a malfunction in NK cell abilities is a key factor in tumor cells evading the immune system. While NK cells are essential in controlling HCC, their efficacy appears to wane in advanced stages of the illness, suggesting that enhancing the performance of impaired NK cells might boost the eradication of cancerous cells [31].

Several previous studies showed that Sorafenib has beneficial effects on the anticancer effector functions of NK cells [32], [33], [34], [35], while other studies reported that Sorafenib may inhibit NK cell effectiveness [36], [37], [38], [39]. In our earlier research, we investigated how Sorafenib and NK cells, separately or together, could be effective against HCC [40], [41], [42]. Nevertheless, further investigation is required to elucidate specific molecular elements and signaling pathways regarding the integration of NK cells and Sorafenib. In summary, our previous research findings indicate that while Sorafenib and NK cells demonstrate strong anti-cancer properties individually, but their combined therapeutic didn’t show the anti HCC tumor potential. The optimization of these two agents for combinational therapy of HCC cancer is complex and requires more research and detailed investigations of signaling pathway. The current research, for the first time, sought to examine the impact of simultaneous intervention of NK cells and Sorafenib on HCC tumors by focusing on the essential genes in signaling pathways involved in HCC tumor progression.

## 2 Materials and methods

### 2.1 Reagents

We mixed Sorafenib powder obtained from American LC LAB Company in a 1 ml of a mixture containing 12.5 % ethanol, 12.5 % Cremophor, and 75 % water in a ratio of 1:1:6 to achieve a concentration of 21.6 mg/ml. Based on the stock solution’s concentration, a desired dose of 30 mg/kg was administered. The precise volume of this solution for each animal was calculated individually according to its measured body weight to ensure accurate dosing at 30 mg/kg. This dosage is consistent with effective concentrations reported in human studies (400 mg, twice daily) [43]. Flow cytometry was utilized with anti-CD56 (PE, EXBIO, Czech Republic) and anti-CD3 (FITC, Beckman Coulter, US) antibodies to detect NK cells. Recombinant interleukin-2 and interleukin-15 from eBioscience (US) were used to activate and expand NK cells.

### 2.2 Cell culture

The Iranian National Center for Genetic and Biologic Resources in Tehran provided HepG2 human HCC cell lines, which were cultured in DMEM with high glucose, 10 % fetal bovine serum, 100 U/ml penicillin, and 100 μg/ml streptomycin under standard conditions (37 °C, 5 % CO2, and 95 % humidity). The NK cells were extracted from peripheral blood mononuclear cells (PBMCs) of healthy individuals utilizing the Miltenyi Biotec NK cell Isolation Kit and magnetic associated cell sorting (MACS) columns through negative selection method, subsequently cultured in SCGM medium (CellGenix, Freiburg, Germany) with anti-CD3 Antibody (OKT3) (10 ng/ml), penicillin (100 IU/ml), 10 % FBS, and streptomycin (100 mg/ml) along with irradiated autologous PBMCs as feeder layer. In a prior investigation, NK cell activation was triggered by hrIL-2 (1000 IU/ml) and hrIL-15 (10 ng/ml) [41]. In each experiment, the purity of the enlarged human NK cells (CD56 +, CD3-) was consistently above 95 %, as assessed by flow cytometry.

### 2.3 Xenograft model and treatment

The athymic male nude (NU/NU; C57BL/6) mice, aged 6 weeks, were obtained from the Pasteur Institute of Iran. The mice were all kept and cared in a controlled environment with no pathogens, each in their own ventilated cage, at 23 °C with 65 % humidity. In accordance with the findings outlined in our earlier research efforts [40], [44], HCC tumors were induced in mice by injecting a mixture of 1 × 10^7^ human HepG2 cells in 100 μl serum-free medium and 100 μl matrigel (in a 1:1 ratio) into both sides of the mouse (six tumors per group). The tumor's size was measured three times weekly with a vernier caliper and calculated using a standard formula of ((length × width^2^)/2).

Therapy started on the twelfth day following the injection of HepG2 cells when the tumor grew to a size larger than 200 mm^3^. The therapy groups were split into four random groups: (a) control with vehicle, (b) Sorafenib, (c) NK cells (5 × 10^6^ cells/100 μl/mouse), and (d) Sorafenib combined with NK cells. Each day, Sorafenib was administered via injections into the peritoneum at a dose of 30 mg/kg, while activated human NKCs were injected straight into the tumors on days 12 and 19 post tumor implantation. All control mice were injected with the carrier solution simultaneously. After following a 40-day monitoring period, the mice were sacrificed after blood samples and tumors were gathered under anesthesia with ketamine/xylazine for the examination of biochemical factors and signaling proteins. The serum levels of liver and kidney biochemical factors and also IFN-γ and TNF-α were measured to assess the safety of the treatment. After measuring the tumor dimensions, total RNA was extracted from the HCC xenograft tumors and evaluated to study the signaling pathways. The animal euthanasia was performed by spinal cord injury that must first cause rapid loss of consciousness by disrupting the central nervous system. According to American Veterinary Medical Association (AVMA) guideline, the thumb and index finger are placed on either side of the neck at the base of the skull or, alternatively, a rod is pressed at the base of the skull with the animal laying on a table surface. With the other hand, the base of the tail is firmly and steadily pulled to cause separation of the cervical vertebrae and spinal cord from the skull. The research received approval from the Institutional Ethics Committee (ethical approval no. IR.MUQ.REC.1399.272).

### 2.4 Tumor growth analysis

The therapeutic effectiveness of NK cells and Sorafenib (either separately or together) against xenograft HCC tumor caused by human HepG2 cell was evaluated by assessing the tumor growth in nude mice as follows: two times a week for four weeks after treatment with a vernier caliper and using a standard formula of ((length × width^2^)/2).

### 2.5 Assessment of in vivo toxicities

#### 2.5.1 Examination of of liver and kidney biochemical factors

The safety of cellular treatment in living organisms was evaluated by analyzing biochemical markers in the liver and kidney. In order to accomplish this objective, blood samples were centrifuged at a speed of 1500 g for a period of 10 min to isolate the serum. The AST, ALT, BUN, and Cr levels were assessed with a biochemical analyzer made by Mindray in China.

### 2.6 Quantitative real-time PCR assay

Levels of signaling factors related to HCC tumor advancement were assessed through qRT-PCR, including proliferative factors (BIRC5, CCDN1, Myc, OCT4, β-catenin), angiogenic factors and tyrosine kinase receptors (VEGFR-2, VEGFR-3, PDGFR-β), apoptosis related factors (Mcl-1, Bcl-2) and factors associated with the anti-tumor effect of NK cells (perforin, granzyme-B, IFN-γ and TNF-α genes). HCC tumor samples were obtained from different treated groups and total RNA was isolated using a total RNA extraction kit (Takara, Japan) according to the manufacturer's instructions. The Takara cDNA Synthesis Kit from Japan was utilized to generate cDNA from 1 µg of RNA with a 260/280 ratio of approximately 2.

The quantification of gene expression was done using qRT-PCR with ABI-7000 detection system thermal cycler by Applied Biosystems in the USA, with SYBR Premix Ex Taq (Takara, Japan), 10 Pm of each primer, 100 ng cDNA (2 µl), SYBR Green I Master Mix (2X) (BioFact, Korea), and nuclease-free H_2_O in a total volume of 20 μl. Each gene underwent identical temperature conditions, including an initial denaturation at 95 °C for 5 min followed by 40 rounds at 95 °C for 30 s (initial denaturation), 60 °C for 30 s (annealing), and 72 °C for 30 s (extension), plus one cycle of a melting curve at 95 °C for 15 s, 60 °C for 1 min, and 95 °C s for 15 s. The qRT-PCR tests were performed twice, and the findings were evaluated using the comparative Ct, 2^–ΔΔCT^ method, and standardized with Glyceraldehyde 3-phosphate dehydrogenase (GAPDH) mRNA as the internal control.

### 2.7 Statistical analysis

Data are presented as mean ± standard deviation (SD). The Shapiro–Wilk test was used to check normality. If the data followed a normal distribution, one-way ANOVA was applied, followed by Tukey’s post hoc test for multiple comparisons. If the data were not normally distributed, the Kruskal–Wallis test was used, followed by Dunn’s multiple comparison test. The differences in variances between the two groups were examined using the Student's t test for independent means. GraphPad Prism software evaluated the data, and the 2^–ΔΔCT^ method was used to determine the relative levels of transcripts. The importance of the statistical differences was established by the p-values being below 0.05 (indicated as p < 0.05 *, p < 0.01 **, and p < 0.001***).

## 3 Results

### 3.1 Analysis of tumor growth

**Fig. 2:**
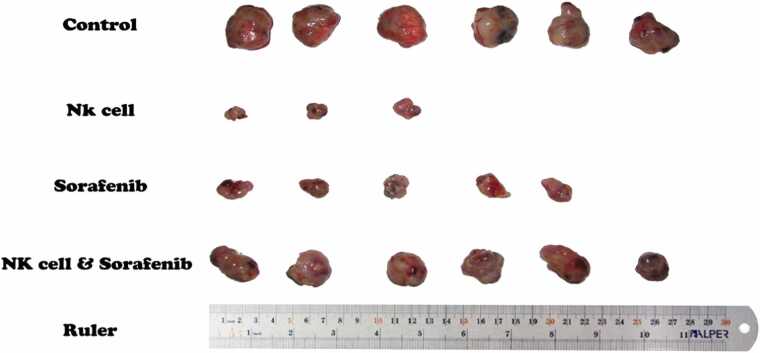
The xenograft HCC tumors removed from differentially treated nude mice on the 40th day of the study.

As illustrated in Fig. 2 and Table 1, the significant inhibition of tumor growth was observed after individual application of NK cells compared to the vehicle-treated animals. Half of the tumors treated with activated NK cells were completely disappeared. Also, Sorafenib administration alone significantly inhibited HCC tumor growth compared to control group. However, combined therapy with NK cells and Sorafenib (at the mentioned dosage) induced no synergic effect against HCC tumor growth and the tumor volumes of this group did not show significant differences compared to the control group.

**Table 1:** The mean size of HCC tumors in different treated xenografts in nude mice since the first day until 28th day after start treatment. The results were evaluated statistically using a one-way ANOVA analysis with repeated measures followed by Tukey post hoc, n = 6 tumors at the beginning of the study, of which 3 tumors in the NK cell-treated group and 1 tumor in the sorafenib-treated group had disappeared by the end of the study.

	Day 1	Day 4	Day 7	Day10	Day13	Day16	Day19	Day22	Day25	Day28
Control	237.82	236.68	202.5	226.84	220.7	272.71	331.9	402.5	460.6	505.19
NK cell	212.48	194.78	146.75	177.08	75.12	57.36	53.74	49.5	37.19	17.1
p-value	> 0.05	> 0.05	> 0.05	> 0.05	< 0.05	< 0.01	< 0.01	< 0.05	< 0.01	< 0.05
Sorafenib	180.33	178.61	178.54	177	142.33	132.05	120.93	95.67	75.83	68.59
p-value	> 0.05	> 0.05	> 0.05	> 0.05	> 0.05	< 0.05	< 0.05	< 0.05	< 0.05	< 0.05
NK Cell & Sorafenib	244.53	182.17	218.63	177.35	175.6	223.97	279.78	298.72	315.38	315.56
p-value	> 0.05	> 0.05	> 0.05	> 0.05	> 0.05	> 0.05	> 0.05	> 0.05	> 0.05	> 0.05

Note: The mean tumor size significantly decreased in individually NK cell or Sorafenib treated group, but combined trated tumors didn’t show meaningful differences compared to control group

The average amount of tumor size in each group represented in Table 1. The one-way ANOVA and then Tukey post hoc analysis revealed the significant inhibition of tumor growth in individual NK cell treated group compared to the vehicle-treated animals on days 13, 16, 19, 22, 25 and 28. Also, Sorafenib administration alone significantly inhibited HCC tumor growth compared to control group on days 16, 19, 22, 25 and 28.

### 3.2 Analysis of biochemical factors

The levels of AST, ALT, BUN, and Cr (U/L) were all within the normal ranges, indicating no notable disparity between the groups as indicated in Table 2.

**Table 2:** Evaluating the safety of cell therapy through monitoring plasma levels (U/L) of biochemical markers for liver and kidney function (ALT, AST and BUN, Cr) in different treated mouse models of HCC.

	Biochemical markers (U/L)			
Group	AST	ALT	BUN	Cr
Control	88.5	80.5	95.5	1.3
Sorafenib	82.5	60.5	65.5	1.4
NK cell	71.5	74	78.5	1.2
Sorafenib & NK cell	86.5	78.5	77	1.55

Note: There was no significant distinction observed between the control and treatment groups. Mean ± SD is used to present all values. One-way ANOVA analysis followed by Tukey post-hoc test. *ALT* alanine aminotransferase, *AST* aspartate aminotransferase, *BUN* blood urea nitrogen, *Cr* creatinine.

### 3.3 qRT-PCR analysis

#### 3.3.1 Tyrosine kinase receptors

**Fig. 3:**
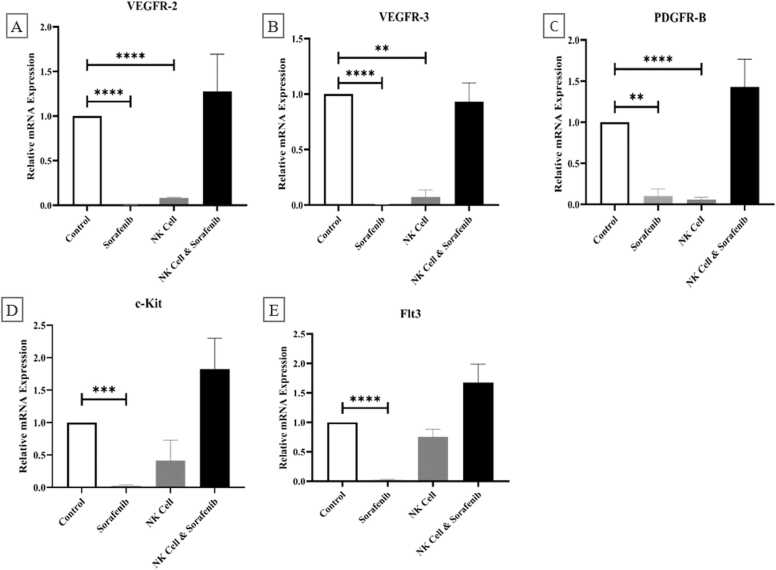
Assessing the expression levels of important tyrosine kinase receptors (VEGFR-2, VEGFR-3, PDGFR-B, c-Kit, Flt3) during HCC tumor initiation compared to Sorafenib targeted therapy in four treated groups (Control, Sorafenib, NK cell, Sorafenib & NK cell). Treatment with either NK cell alone or Sorafenib resulted in a substantial reduction in receptor expression levels. The NK cell & Sorafenib treated group had the highest levels of expression for all factors, with no discernible differences compared to the control group. The Student's t test was utilized to assess the disparities between the two groups. Statistical variances were considered notable at p-values of less than 0.05 (*), 0.01 (**), 0.001 (***), and 0.0001 (****).

**As** demonstrated in the Fig. 3**,** the statistical analysis has shown that the average levels of Tyr kinase receptors were notably decreased in groups treated with only Sorafenib compared to control group (VEGFR-2 (p-value= 0.00116), VEGFR-3 (p-value= 0.00317), PDGFR-B (p-value= 0.10017), c-Kit (p-value= 0.02484), Flt3 (p-value= 0.02367)). Also the expression levels of some genes in the NK cell treated group showed statistically significant decreased compared to control group (VEGFR-2 (p-value=.000017), VEGFR-3 (p-value= 002247), PDGFR-B (p-value< 0.00001)). However, c-Kit (p-value= 0.92642) and Flt3 (p-value= 0.75282) gene expression did not show a significant decrease in the NK cells treated group in comparison to control group. Nevertheless, the groups that received combination treatment did not show any significant differences in the levels of receptor Tyr kinases compared to the control group (VEGFR-2 (p-value= 1.27581), VEGFR-3 (p-value= 0.93204), PDGFR-B (p-value= 1.42839), c-Kit (p-value= 1.82369), Flt3 (p-value= 1.67600)).

#### 3.3.2 Ras/Raf/MEK/ERK and JAK/STAT signaling pathway

**Fig. 4:**
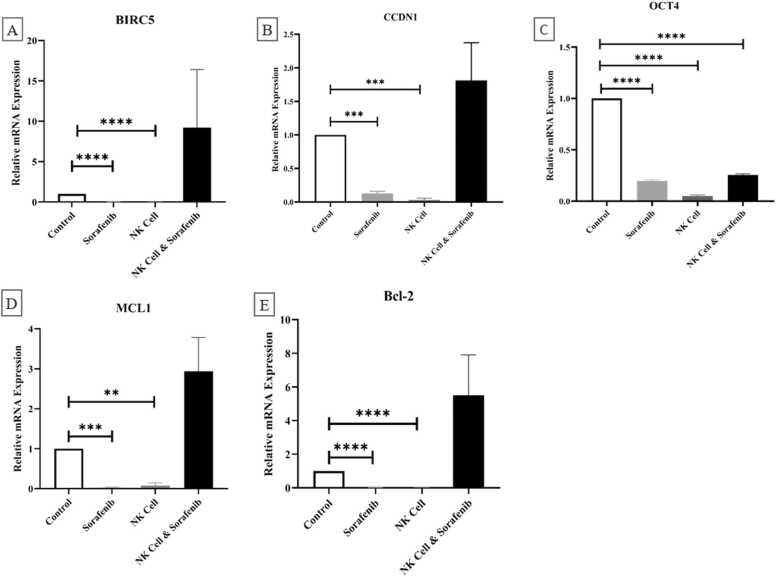
The qRT-PCR analysis was utilized to assess the expression levels of important downstream target genes of the Ras/Raf/MEK/ERK and JAK/STAT signaling pathways (BIRC5, CCDN1, OCT4, Bcl-2, and Mcl-1) in xenograft HCC tissues following various treatments. Treatment with either NK cell alone or Sorafenib resulted in a notable decrease in the levels of these genes linked to HCC cell proliferation, viability, and cell death. However, there were no significant alterations in the gene expression of any genes, except for OCT4, in the NK cell & Sorafenib treated group when compared to the control group. Student's t test for independent means was used to analyze the variances between the two groups. Emphasis was placed on the significance of statistical variations with p values below 0.05 (*), 0.01 (**), 0.001 (***), and 0.0001 (****).

A significant decrease in the relative expression levels of important target genes in the Ras/Raf/MEK/ERK and JAK/STAT signaling pathways was observed in the monotherapy group treated with Sorafenib ((BIRC5 (p-value= 0/00989), CCDN1 (p-value= 0/12826), Bcl-2 (p-value= 0/00939), Mcl-1 (p-value= 0/02335)) or NK cells ((BIRC5 (p-value= 0/008438), CCDN1 (p-value= 0/03014), Bcl-2 (p-value= 0/02653), Mcl-1 (p-value= 0/07898)) compared to control group (Fig. 4). However, there was no significant decrease in the expression level of these genes in the group that received combined treatment compared to the control group (p-value>0.05). The levels of OCT4 gene expression significantly decreased in all treated groups in comparison to the control group (p < 0.0001).

#### 3.3.3 Wnt/β-catenin signaling pathway

**Fig. 5:**
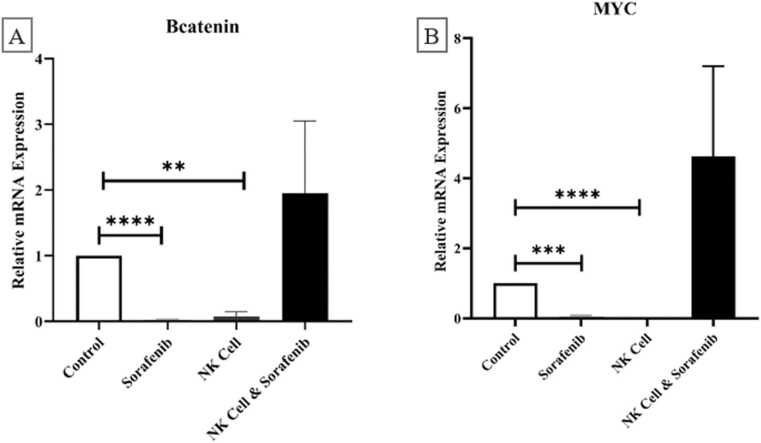
Evaluation of the expression of the key target gene of the Wnt/β-catenin signaling pathway (MYC), involved in tumor progression. The findings indicated a notable rise in β-catenin and MYC gene expression in either the NK cell or Sorafenib group when used as a standalone treatment. However, the combined therapy of NK cell and Sorafenib resulted in increased β-catenin and MYC gene expression levels, although the difference was not statistically significant. The Student's t test for independent means was employed to examine the differences in variances between the two groups. Statistical significance was determined for variances with p values less than 0.05 (*), 0.01 (**), 0.001 (***), and 0.0001 (****).

The statistical analysis presented a notable reduction in the relative amounts of β-catenin and its target gene MYC in HCC tumors after treatment with Sorafenib (β-catenin (p-value= 0.02724), MYC (p-value= 0.04531)) or NK cells ((β-catenin (p-value= 0.07618), MYC (p-value= 0.00219)) compared to control group in Fig. 5. Nevertheless, combining Sorafenib and NK cells did not show significant impact on the expression levels of β-catenin (p-value= 1/95024) and MYC (p-value= 4/63545) compared to the control group.

#### 3.3.4 The pathways of NK cell effector function against HCC tumors

**Fig. 6:**
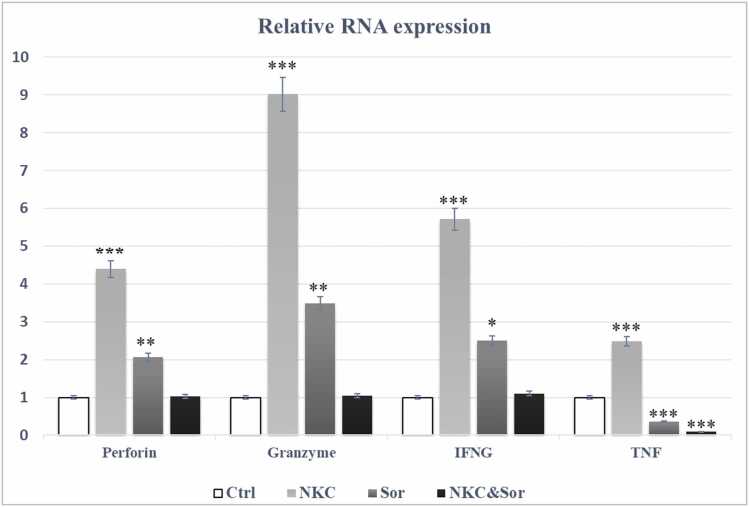
Evaluation of the expression of the key NK cell effector function genes (Perforin, Granzyme-B, IFN-γ and TNF-α) in different treated group. Expression of mRNA was quantified by real-time PCR and normalized to GAPDH gene, n = 6 tumors for each group. Ctrl control, Sor Sorafenib, NKC NK cells, NKC & Sor combination of NK Cells and Sorafenib. The Student's t test for independent means was employed to examine the differences in variances between the two groups. Statistical significance was determined for variances with p values less than 0.05 (*), 0.01 (**), 0.001 (***), and 0.0001 (****).

As shown in Fig. 6**,** the statistical analysis indicated a significant up-regulation of all perforin, granzyme-B, IFN-γ and TNF-α genes in the NK cell-treated group compared to control groups (p < 0.001). Also Sorafenib significantly induced the expression level of perforin, granzyme-B and IFN-γ compared to control group (p < 0.01, p < 0.01 and p < 0.05 respectively), but led to meaningful decrease of TNF expression (p < 0.001). The expression levels of perforin, granzyme-B and IFN-γ showed no significant difference in the combined treated group compared to control group and also TNF-α significantly down regulated in this group, indicating no synergic effect of NK cell and Sorafenib against HCC and the inhibitory effect of Sorafenib on the effector function of NK cells via cytokine production and degranulation.

## 4 Discussions

HCC is a prevalent issue worldwide and ranks third in cancer-related mortality globally [45]. HCC is a complex malignancy with limited treatment options, particularly for advanced stages. Sorafenib, a multi-kinase inhibitor, is a standard therapeutic agent for HCC, but its efficacy is often hampered by resistance and moderate response rates. Although Sorafenib is successful in treating HCC, its low response rate and adverse events make it unsuitable [23]. Hence, it has been proposed that the effectiveness of treatment for HCC could be improved by combining Sorafenib with other therapies such as PI3K/mTOR inhibitors and STAT3 blockers, offer promising strategies to enhance Sorafenib efficacy and overcome resistance [46], [47], [48], [49]. Over the past ten years, numerous researchers have concentrated on harnessing adoptive NK cells for immunotherapy in HCC, because of their powerful anti-tumor properties. NK cells play a significant role in inhibiting the growth of HCC. However, NK cells can also become dysfunctional in the HCC microenvironment, leading to decreased anti-tumor activity because of receptor imbalance, tumor-derived inhibitory factors, NK Cell exhaustion or tumor evasion mechanisms [50]. Approaches like adoptive transfer of NK cells, cytokine-induced activation of NK cells, and strategies to reverse NK cell exhaustion are being explored as potential therapies for HCC. Combining NK cell immunotherapy with conventional therapies like chemotherapy or other immunotherapies may offer a more effective approach to treating HCC [30]. Despite the effectiveness of combination therapy with Sorafenib and NK cells against HCC tumors, there are contradictory findings on how Sorafenib and NK cells may influence each other [36], [37], [51], [52]. This response showed the importance of exploring the signaling pathways affected by Sorafenib and NK cells in HCC, focusing on cellular and molecular biology. We investigated how effective combining Sorafenib and NK cells in a co-injection is, compared to injecting them separately, in a xenograft mice model of HCC. With the importance of signaling pathways and molecular targeted therapies in cancer research [7], we examined the impact of NK cells on the signaling pathways linked to HCC progression affected by Sorafenib through the evaluation of targeted gene expression downstream.

As shown in Fig. 2 and Table 1, NK cells had the greatest effect in suppressing HCC tumor growth, and half of the NK cells treated tumors were completely destroyed. These results are consistent with the results of previous studies regarding the antitumor properties of NK cells against HCC tumors [53]. Also in accordance with previous studies, the tumor growth of Sorafenib treated group was significantly inhibited compared to the control group [54]. However, the combinational treated tumors showed no significant difference compared to control group, indicating no synergic effect of NK cells and Sorafenib against HCC. The results of liver and kidney function by measuring the levels of AST, ALT, BUN, and Cr are shown in Table 2, these factors were examined in our study to ensure the safety of our interventions and none of which showed any significant signs of toxicity.

Sorafenib hinders the inhibition of tyrosine kinase receptors such as VEGFR-2, VEGFR-3, PDGFR-β, EGFR, c-Kit, and Flt3, which ultimately impedes the proliferation of tumor cells and angiogenesis [20], [25]. In this research, the levels of VEGFR-2, VEGFR-3, PDGFR, c-Kit and Flt3 were assessed in tumor tissues treated with various methods (at the specified dose and duration). As depicted in Fig. 3, Sorafenib exhibited the most potent anti-angiogenic impact through the reduction of VEGFR-2 and VEGFR-3 expression. Both NK cells and Sorafenib independently caused a decrease in these receptors, however, their combination did not yield any notable difference in comparison to the control group. Additionally, a significant reduction in PDGFR-β levels was observed in NK cells or Sorafenib treated groups in comparison to control and combination treated groups, resulting in the inhibition of tumor growth. These results indicate that in the designated dose and timing of treatment, Sorafenib and NK cells hinder each other's ability to inhibit cell proliferation. Moreover, the levels of c-Kit and Flt3 receptors' expressions were evaluated in four separate groups following various treatments. While Sorafenib showed significant effectiveness at blocking the expression of c-Kit and Flt3 genes, in the combinational treated group its efficacy decreased when NK cells were included in the treatment and showed no meaningful difference compared to control group (Fig. 3).

These results are consistent with those of previous studies which showed Sorafenib reduces the number and activity of NK cells in HCC tumors by inhibiting ERK phosphorylation and decreasing plasma levels of MCP-1 and IFN-γ [55]. Also, the finding of another study showed that Sorafenib disrupted the AKT, MEK, and ERK signaling pathways, impairing NK cell activation [56]. The results of our study are in line with the finding of several previous studies that showed Sorafenib, a multi-tyrosine kinase inhibitor, has off-target effects on host immunity, particularly by suppressing NK cells, which may contribute to increasing tumor growth and metastasis in HCC models.

It was reported that Sorafenib primarily affects the proliferation and function of NK cells by blocking the PI3K/AKT and ERK pathways, rather than directly impacting the JAK/STAT3 pathway [37]. Other study also highlighted that Sorafenib inhibits NK cell cytotoxicity and IFN-γ production through impaired PI3K and ERK phosphorylation, affecting NK cell reactivity [38].

The expression levels of the most important target genes of Ras/Raf/MEK/ERK and JAK/STAT signaling pathways including OCT4, Bcl-2, Mcl-1, cyclin D1 (CCND1), and surviving (BIRC5) in different treated groups have been illustrated in Fig. 4. There was a significant reduction in the expression levels of BIRC5 and CCDN1 in both groups that received Sorafenib or NK cells.

However, the cooperation between Sorafenib and NK cells showed no significant difference comparing control group. Additionally, the individual use of Sorafenib or NK cells led to a noteworthy decrease in the expression of anti-apoptotic Mcl-1 and Bcl-2 genes. However, in the group that received both treatments, this effect was limited and did not differ significantly from the control group. This finding suggests that NK cells and Sorafenib may hinder each other's ability against HCC tumor growth by modulating JAK/STAT, PI3K/AKT/mTOR, Ras/Raf/ERK/MEK or Wnt/β-catenin pathways, thereby affecting the expression of Bcl-2, Mcl-1, cyclin D1, and survivin levels. Also, as demonstrated in Fig. 4, both Sorafenib and NKC, whether used alone or together, resulted in significantly decreased of OCT4 expression compared to control group.

These findings are consistent with previous studies that showed Sorafenib inhibit the JAK/STAT signaling pathway and regulate the levels of pSTAT3 and its downstream genes necessary for cellular activities like metabolism, growth, angiogenesis, and survival, such as Bcl-2, Mcl-1, cyclin D1, and survivin by enhancing the amounts of SHP-1 and SHP-2 phosphatase [20], [57]. Furthermore, previous research has shown that OCT4, as a different signaling protein, can stimulate the proliferation of HCC cells by increasing the levels of BIRC5 and CCND1 expressions [58]. On the other hand, studies have shown that Sorafenib can induce resistance in cancer cells, and in these resistant cells, the expression of OCT4 (a marker for cancer stem cells) is often increased [59].

The exact mechanism of how NK cells affect OCT4 expression in HCC is still being researched, but it's likely related to their ability to kill cancer cells and modulate the tumor microenvironment. The results of combined treated groups can be justified by finding of one study that showed Sorafenib inhibits the JAK-STAT pathway by decreasing phosphorylation of STAT1 and STAT5 in response to IFN-α and IL-2, respectively, leading to reduced NK cell function, including diminished production of cytokines like IFN-γ and impaired proliferation [56].

The Wnt/β-catenin pathway, found in at least one-third of HCC tumors, is considered one of the most difficult to inhibit. This pathway controls the transcription of specific genes such as Cyclin D1, c-myc (MYC), and Survivin, assisting in the advancement of the cell cycle and the prevention of apoptosis [5], [60]. Fig. 5 showed that Sorafenib or NK cells led to down-regulation of β-catenin and MYC genes, while in combination increased the expression levels of these genes, suggesting interference in their anti-tumor effects. In line with these results, the other study has been reported that Sorafenib could potentially regulate Wnt signaling by reducing the expression of β-catenin [61]. Also, this result may be related to the fact that MYC is required for mature NK cell development [62]. While the direct impact of NK cells on β-catenin and MYC expression is less explored, their role in modulating the tumor microenvironment can influence the effectiveness of Sorafenib and the overall tumor response [63].

For evaluation of the Sorfenib effect on the NK cell effector function in combinational therapy of HCC, the expression levels of most important factors including TNF-α, IFN-γ, Perforin, Granzyme was measured in the xenograft HCC tumors. As shown in Fig. 6**,** the mentioned factors were significantly upregulated in the NK cell treated group. But unexpectedly the expression levels of these factors were decreased in combination treated groups to the extent that did not show any significant difference with the control group. These findings confirm the inhibitory effect of Sorafenib on the anti tumor effect of NK cells in combination therapy in the specified doses mentioned.

The observed loss of efficacy when combining Sorafenib with NK cells in treating HCC can be attributed to several interrelated factors. While Sorafenib is a standard treatment, it has been shown to suppress NK cell activity, which may counteract the intended synergistic effects of the combination therapy. As reported in recent study, Sorafenib significantly reduces the number and activity of NK cells in tumor-bearing models, leading to decreased levels of critical cytokines like IFN-γ and MCP-1 [55]. Also, these findings are align with Zhang *et al*.'s study, suggesting that Sorafenib inhibits NK cell proliferation and cytotoxicity by blocking the PI3K/AKT pathway and impairing ERK phosphorylation, which are essential for NK cell function [37].

In addition, the tumor microenvironment in HCC is often immunosuppressive, which can hinder NK cell expansion and persistence, limiting their effectiveness even when combined with Sorafenib [51]. These results are consistent with previous research that showed Sorafenib hinders the effectiveness of NK cells when given together at certain specified levels and schedules [40]. In addition, Krusch *et al*. also have reported that Sorafenib inhibits NK cell activity, reducing their cytotoxicity and IFN-γ production, which are crucial for antitumor immunity [38].

Accordingly, while the combination of Sorafenib and NK cells shows promise, the inherent challenges of NK cell suppression and tumor microenvironmental factors may necessitate additional strategies to optimize treatment efficacy. Several studies suggest that enhancing NK cell functionality through cytokine activation or combining Sorafenib with immune checkpoint inhibitors, like anti-PD-L1, may improve therapeutic outcomes by overcoming the immunosuppressive effects of Sorafenib [64]. A recent study revealed that combining IL-12/IL-18-pretreated NK cells with Sorafenib improved the inhibition of HCC tumor growth [65].

In conclusion, Sorafenib, a tyrosine kinase inhibitor, is known to affect various signaling pathways. There is increasing evidence that Sorafenib can regulate the function of immune cells, particularly NK cells against HCC tumors, particularly in combination treatments. This reduction is primarily due to Sorafenib's immunosuppressive effects, which diminish NK cell function and their ability to lyse tumor cells. In combination therapies, the negative impact on NK cell efficacy may be exacerbated, as Sorafenib can modulate the immune response, further impairing NK cell activation and expansion. Understanding these dynamics is crucial for optimizing treatment strategies, as identifying effective combination therapies could help mitigate Sorafenib's adverse effects on NK cells, thereby enhancing overall anti-tumor responses. The mechanism by which Sorafenib affects NK cell function is not yet comprehensively clarified, suggesting that varying doses could potentially influence NK cell efficacy against HCC tumors, warranting further investigation into optimal dosing strategies. Therefore, careful consideration of NK cell-mediated cytotoxicity is essential when developing treatment regimens for HCC that include Sorafenib and a comprehensive understanding of the mechanism is necessary in order to optimize the combination of Sorafenib and NK cell-based immunotherapy for the treatment of HCC.

## 5 Conclusions

Sorafenib and NK cells modulate multiple signaling pathways in HCC, including Ras/Raf/MEK/ERK, NF-κB, PI3K/AKT/mTOR, Wnt/β-catenin and JAK/STAT pathway. These pathways are critical for tumor growth, immune evasion, and drug resistance. Combination therapies targeting these pathways offer promising strategies to enhance Sorafenib and NK cell efficacy and overcome drug resistance. In this study, we investigated how Sorafenib and NK cells impact the key signaling pathways that contribute to the development of HCC tumors in human HCC xenograft mouse models, either individually or in combination. Analyzing the relationship between Sorafenib and NK cells at a molecular level demonstrated that when used together, they can impede the antiproliferative, antiangiogenic, and apoptotic effects on HCC tumors. Our findings suggest that Sorafenib may not be the optimal option for cancer treatment when used in conjunction with NK cells for managing HCC in the described manner. Nevertheless, we suggest further mechanistic studies that could elucidate the precise interactions between Sorafenib and NK cells that lead to this non-synergistic outcome. Also, more research is needed to examine how different doses of Sorafenib and NK cells interact in combined treatment for HCC, since the effects of Sorafenib on NK cell functions differ depending on dosage and timing.

## Declarations

• **Ethical Publication Statement:** We confirm that we have read the Journal’s position on issues involved in ethical publication and affirm that this report is consistent with those guidelines.
• **Funding:** Not applicable.
• **Disclosure of Conflicts of Interest:** None of the authors have any conflicts of interest to disclose.
• **Ethics approval and consent to participate:** The experimental methods followed the the National Institutes of Health (NIH) guidelines for Lab animal care and were approved by Qom University's Institutional Ethical Committee (IR.MUQ. R.E.C.1399.272).

## CRediT authorship contribution statement

**Javad Verdi:** Validation, Conceptualization. **Nima Beheshtizadeh:** Writing – review & editing, Visualization, Validation, Investigation. **Masoumeh Dolati:** Writing – original draft, Software, Methodology, Investigation, Formal analysis. **Amir Hossein Kheirkhah:** Software, Resources, Methodology, Formal analysis, Data curation. **Faezeh Hosseinzadeh:** Writing – original draft, Visualization, Software, Resources, Project administration, Funding acquisition, Conceptualization. **Tahereh Komeili movahhed:** Writing – original draft, Visualization, Software, Resources, Methodology, Data curation.

• **Acknowledgments:** The authors would like to thank to the MUQ and TUMS for its support.
• **Consent for publication:** Not applicable.
• **Availability of data and materials:** All data and materials are available from the corresponding author.

## Data Availability

Data will be made available on request.
